# Supplementary material for: Screening for undiagnosed non-alcoholic fatty liver disease (NAFLD) and non-alcoholic steatohepatitis (NASH): A population-based risk factor assessment using vibration controlled transient elastography (VCTE)
Source: PLoS One. 2021 Nov 30;16(11):e0260320. doi: 10.1371/journal.pone.0260320 (PMC8631660; doi:10.1371/journal.pone.0260320)
Supplement: S1 File — Demographics and past medical history survey questions. (PDF) [file pone.0260320.s001.pdf]

S1 Survey. Demographics and past medical history survey questions.

The following information was collected from each participant that successfully completed the pre-screening round.

Demographics:
- Sex
- Age
- Race/ethnicity
- Height
- Weight

Metabolic Syndrome:
Subjects will use a form to check YES or NO for comorbid components of the metabolic syndrome they may or may not have:
- Obesity/overweight
- Diabetes mellitus
- Hypertension
- Hypercholesterolemia
- Hypertriglyceridemia
- Gout or elevated uric acid

Other Health Conditions:
Subjects will use a form to check YES or NO for other health conditions they may or may not have:
- Heart disease
- Heart attack
- Arrhythmia
- Stroke
- Arthritis
- Osteopenia/osteoporosis
- Hypothyroidism
- Low Vitamin D
- Low testosterone
- Menopausal state
- Inflammatory bowel disease (ulcerative colitis or Crohn's disease)
- Cancer
    - Skin: melanoma
    - Skin: basal cell or squamous cell cancers
    - Other types of cancers: lymphoma, leukemia, head or neck, thyroid, brain, breast, lung, stomach, colon, liver, ovary, uterus, prostate, bone

Diet:
Subjects will fill out a form using a 5-point scale of the frequency of their average weekly dietary intake of specific food types (A. 0 days; B. 1-2 days; C. 3-4 days; D. 5-6 days; E. 7 days per week)
- Proteins: eggs, cheese, beans, fish, chicken, turkey, beef, pork
- Starchy carbohydrates: breads, rolls, pasta, cereals, potatoes, rice, corn, peas, lima beans
- Non-starchy vegetables: lettuces, kale, carrots, green beans, broccoli, peppers, onions, cucumber
- Fats: butter, margarine, avocado, oils (olive, safflower, cottonseed, peanut, sesame, walnut)
- Nuts: peanuts, walnuts, cashews, almonds, macadamias, peanuts
- Sugars or Syrups: sugar, high fructose corn syrup (soda), maple or corn syrups

- Fast food meals
- Eating out at/getting takeout from restaurants

Exercise:
Subjects will fill out a form using a 5-point scale of the frequency of their average weekly exercise: (A. 0 days; B. 1-2 days; C. 3-4 days; D. 5-6 days; E. 7 days per week)

- Cardiovascular: Brisk walking, jogging, biking, treadmill, stationary bike, stair stepper, elliptical, swimming laps
- Strength: Weightlifting, resistance machines or cords

Alcohol:
Subjects will fill out a form quantifying their average weekly total of alcoholic drinks:
(1 drink = 12 oz beer, 6 oz wine, 1.15 oz hard liquor, 3 oz brandy/sherry liqueur)

- None
- 1-2 drinks/week
- 3-4 drinks/week
- 5-6 drinks/week
- 7-8 drinks/week
- 9-10 drinks/week
- 11-12 drinks/week
- 13-14 drinks/week
- 15-20 drinks/week
- 21 or more drinks/week

Other Liver Diseases:
Subjects will check YES or NO for other liver-related diseases they may or may not have:

- Hepatitis B alone
- Hepatitis B with Hepatitis D
- Hepatitis C
- Hepatitis E
- Autoimmune hepatitis
- Primary biliary cholangitis
- Primary sclerosing cholangitis
- Iron overload
- Alpha-1-antitrypsin deficiency
